# Migratory Pathways and Connectivity in Asian Houbara Bustards: Evidence from 15 Years of Satellite Tracking

**DOI:** 10.1371/journal.pone.0020570

**Published:** 2011-06-08

**Authors:** Olivier Combreau, Samuel Riou, Jacky Judas, Mark Lawrence, Frédéric Launay

**Affiliations:** 1 International Fund for Houbara Conservation, Abu Dhabi, United Arab Emirates; 2 National Avian Research Center, Abu Dhabi, United Arab Emirates; 3 Mohamed Bin Zayed Species Conservation Fund, Abu Dhabi, United Arab Emirates; University of California, Berkeley, United States of America

## Abstract

Information on migratory pathways and connectivity is essential to understanding population dynamics and structure of migrant species. Our manuscript uses a unique dataset, the fruit of 103 individual Asian houbara bustards captured on their breeding grounds in Central Asia over 15 years and equipped with satellite transmitters, to provide a better understanding of migratory pathways and connectivity; such information is critical to the implementation of biologically sound conservation measures in migrant species. At the scale of the distribution range we find substantial migratory connectivity, with a clear separation of migration pathways and wintering areas between western and eastern migrants. Within eastern migrants, we also describe a pattern of segregation on the wintering grounds. But at the local level connectivity is weak: birds breeding within the limits of our study areas were often found several hundreds of kilometres apart during winter. Although houbara wintering in Arabia are known to originate from Central Asia, out of all the birds captured and tracked here not one wintered on the Arabian Peninsula. This is very likely the result of decades of unregulated off-take and severe habitat degradation in this area. At a time when conservation measures are being implemented to safeguard the long-term future of this species, this study provides critical data on the spatial structuring of populations.

## Introduction

To what extent do migratory birds from the same breeding population travel to the same wintering grounds, and vice-versa? Setting aside that migratory connectivity [1], [2] has intrigued naturalists for centuries, the degree of connectivity has direct implications for the evolution and conservation of birds [3], [4]. Yet it remains poorly documented for all but a few species.

Knowledge on the extent of migratory connectivity is essential to the understanding of population dynamics [5]. When connectivity is strong, i.e. when most individuals from one breeding population move to the same non-breeding location, seasonal interactions, in population density for example, are likely to be important [6]. If in addition there are geographical partitions in the migratory pathways of both adults and juveniles (i.e. a migratory divide), this is likely to promote divergence between populations and may lead to allopatric speciation [3]. In the case of endangered species, data on migratory pathways and connectivity, along with population genetic data, are essential to delineate biologically relevant management units [4], [7], [8].

The Asian houbara bustard *Chlamydotis macqueenii* is a ground-dwelling bird distributed in the arid lands ranging from the Nile River to the Gobi desert in Mongolia [9], [10]. It is listed as vulnerable on the IUCN red list due to over-hunting and habitat loss to grazing. Human development has also fragmented populations and caused severe declines, particularly in the southern part of the range [11], [12]. Northern populations (Western Kazakhstan to Mongolia), the last stronghold of this species, are migratory and spend the winter in the Middle-East and South-central Asia. They are heavily hunted there during the winter months. Population genetic studies have been carried out in an attempt to identify distinct populations and management units, but both mitochondrial and microsatellite markers have failed to show enough resolution within Central Asia [13]. The migratory pathways of adults from widely separated breeding grounds as well as the dispersal of juveniles may help identify distinct populations, if these exist, as well as patterns of connectivity between breeding and wintering grounds.

Most of the current knowledge on migration routes and wintering areas has been acquired by bird ringing throughout the 20^th^ century [14], [15], [16]. However, the probability of ring recovery is very low and spatially heterogeneous, which usually means that information obtained is of limited spatial detail and sample sizes are small. More recently, ornithologists attempting to quantify links between breeding and wintering grounds have used the geographical distribution of naturally occurring biological markers including molecular markers [17], [18], stable isotopes [19], [20] and trace elements [21]. These techniques have led to important findings but they provide low spatial resolution and have several drawbacks (e.g. the use of molecular markers is dependant on the presence of detectable genetic structure, and stable isotopes require marked contrasts in isotopic signatures between regions and consistent feather moult schedules). For large animals, an alternative method, and certainly the one which provides the best resolution, is satellite telemetry.

Here we use a large dataset consisting of 103 satellite transmitters deployed on Asian houbara bustards throughout Asia and over 15 years in order to provide a better understanding of migratory pathways and connectivity in this species.

## Methods

Between 1995 and 2008, 21 juveniles and 82 breeding adult houbara were caught in Asia and Eastern Arabia and equipped with satellite transmitters. This was done under agreements signed between the National Avian Research Center (NARC) of Abu Dhabi and the authorities of all concerned countries.

In the northern part of the range, migrant birds were captured during the breeding season in five regions distributed over 5000 km of longitude along a line extending from the Caspian Sea in the west to the Gobi desert of China (Tab.1). Further south, adults were also caught in Northern Iran and in Eastern Arabia (Oman and Yemen, Tab.1). This network of sampling locations covered the greater part of the breeding distribution range of migrant houbara populations in Asia and a section of the resident's range. Juveniles were captured in two locations only, Bedpak-Dala in Kazakhstan and the Jungar basin of China (Tab.1).

In all countries except Yemen, adult birds were captured with the use of loop cord snares following methods described in Launay et *al*. [22]. Females were caught at the nest or attracted to their previously captured chicks (maintained in a soft cloth bag). Males were caught on their display site. In Yemen, a number of captures took place outside the breeding season, precluding the use of chicks or displaying sites for snaring. Instead, we caught 5 of 10 birds with disarmed falcons (i.e. trained to fly with a hood protecting their beak and plastic balls glued to their talons). Juveniles were captured by hand at *30–45* days of age when they still have limited flight capabilities. Birds weighing less than 700 g at capture were fitted with VHF radio transmitters and recaptured at a later stage to be equipped with heavier satellite transmitters fixed with an elastic harness. Siblings were released together at the site of capture and close to mother who generally remains in the vicinity during the process.

Satellite transmitters were mounted on birds in a back-pack configuration using Teflon ribbon (Bally Ribbon Mills, USA). The transmitters used in this study were 35 g solar-powered PTT 100, 30 g battery powered PTT 100, and 30/45 g solar-powered GPS-PTT (Microwave Telemetry Inc., Maryland, USA). The load did not exceed 3% of body weight for males, 4% for females and 6% for chicks at the time of deployment. Our experience, notably in re-trapping houbara equipped in earlier years, suggests that these devices do not cause any detectable physical discomfort or change in behaviour.

Solar-powered PTTs worked according to a 10 hours on/21 hours off duty cycle. Battery powered PTTs were set to transmit for periods of 8 hours either every 2 days or every 4 days. Battery powered PTTs deployed prior to 1997 switched off for three months following the first month of deployment to save battery power. This usually took place in late spring and did not affect the tracking of migratory movements. Solar-powered GPS-PTTs provided between 4 and 12 locations per day. We used GPS and best quality ARGOS classes 1, 2 and 3 to detail migration routes, although when these were not available, lower accuracy locations were very occasionally used (representing less than 1% of the data used from ARGOS PTTs).

In this study we only considered the first autumn migration and wintering area following capture. Routes and wintering locations are highly conserved within individuals [23], [24] and therefore including data for more years was of little value to this study. We used ARCGIS (Environmental Systems Research Institute Inc. Redlands, California, USA), Hawth tools [25] and a topographic map (Digital Elevation Model available from http://eros.usgs.gov) to display bird movements. We used ARCGIS to plot bird movements. Wintering areas were delimited using the local convex hull method (LOCOH) described in Getz & Wilmers [26] and implemented in R [27]. This method, based on the creation of minimum convex polygons, is superior to kernel methods when used in situations where the data contain sharp edges or holes (due to topography for instance). We used a linear modeling approach in R to test for the effects of population origin, sex and age (juvenile vs. adult) on distances travelled. Assumptions of normality and homoscedasticity were checked by diagnostic plots. We used likelihood ratio tests (F-tests) to obtain significance levels for factor effects. Model coefficients described the effect of factor levels and t-tests showed whether the coeffficients were different from zero [28].

## Results

We obtained complete data for the first outward migration (or at least six months of data in resident birds) for 79 individuals (29 males, 37 females and 13 juveniles) out of 103 transmitters deployed (Table 1). Other cases included transmitter failure due to premature battery power drainage (particularly common at the onset of the project) and early death of the bird.

**Table 1:** Year and location of capture of Asian houbara bustards fitted with satellite transmitters.

Location of capture			Date	Number of birds harnessed *				
Country	Region	Area		Males	Females	Juv.	Sub-Total	Total
Kazakhstan	Northeast Caspian	Buzachi Peninsula	1995	1 (0)	1 (1)		2 (1)	23 (19)
			1996	3 (2)			3 (2)	
		Mangystau Oblast	2005	6 (6)	6 (5)		12 (11)	
			2006	6 (5)			6 (5)	
	Central	Bedpak-Dala	2007	2 (2)	4 (3)		6 (5)	27 (24)
			2008	2 (2)	13 (12)	6 (5)	21 (19)	
	South Balkash	Tau Kum desert	1995	7 (0)			7 (0)	9 (2)
			1996	2 (2)			2 (2)	
China	Xinjiang	East Jungar Basin	1998	2 (2)	5 (5)		7 (7)	24 (17)
			1999	2 (2)		3 (3)	5 (5)	
			2000			3 (2)	3 (2)	
			2001			6 (1)	6 (1)	
			2002			3 (2)	3 (2)	
	Gansu	Southwest Gobi	2000	4 (2)			4 (2)	4 (2)
Iran	Semnan	North Dasht-e-Kavir	2003	2 (2)	3 (3)		5 (5)	5 (5)
Oman	Central	Jiddat Al Harasis	2002		1 (1)		1 (1)	1 (1)
Yemen	South	Mayfa'a	2002		1 (1)		1 (1)	10 (9)
			2004	1 (1)	1 (1)		2 (2)	
	East Adramawt	Al Durw plateau	2002	1 (1)	1 (1)		2 (2)	
			2005		2 (2)		2 (2)	
			2006		1 (1)		1 (1)	
			2007		2 (1)		2 (1)	
TOTAL				41 (29)	41 (37)	21 (13)	103 (79)	

*In brackets are the numbers of satellite tagged birds that completed at least one outward migration following capture, or survived more than 6 months (resident birds).

### Migration routes

**Figure 1:**
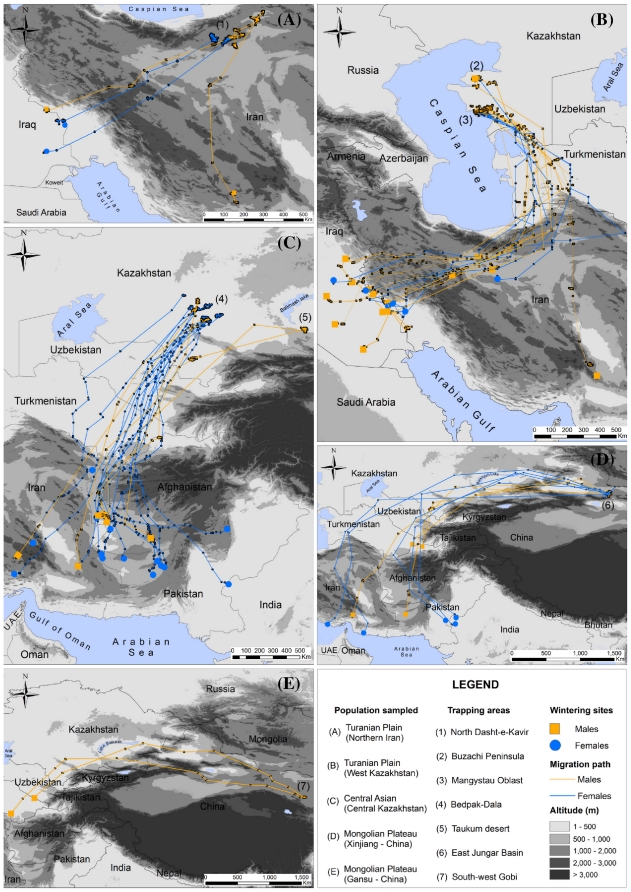
First outward migration routes of Asian houbara bustards captured during the breeding season and followed by satellite tracking.

Houbara breeding in West-Kazakhstan migrated towards the south-east down the coast of the Caspian Sea (Fig. 1B). Upon reaching the southern part of the Caspian Sea, they crossed the Elburz Mountains in North-Iran at an altitude of 1700–2000 m and continued their journey in a south-westerly direction along the northern edge of Dasht-e-Kavir. Birds either stopped on the eastern side of the Zagros Mountains or crossed them at an altitude of 1500–2000 m and wintered around the Iran-Iraq border or further west into Iraq. One bird avoided both mountain chains by following a southerly direction, reaching a wintering area in Kerman province (South-Iran). On average, these birds flew 1885±60 km (Mean ± SE, n = 19) before reaching their wintering grounds.

The five birds captured in North-Iran migrated south-west (Fig. 1A). Four showed the same migration route as the birds caught in West-Kazakhstan, wintering along the Iran-Iraq border. One bird moved north-east during the summer, then, in autumn, it migrated in a more southerly direction than the others. It did not cross the Zagros Mountains and wintered in South-Iran. These birds covered on average 927±63 km (n = 5) on their outward migration.

Birds caught in Central and East Kazakhstan showed overlapping migratory routes. They flew south-west down the western side of the Pamir and then south or south-east around the Hindu-Kush Mountains, avoiding high altitude terrain (Fig. 1C). They wintered south of the Turkmen border and usually south-west of the Hindu-Kush in the Helmand Province of Afghanistan and the Baluchistan Province of Pakistan. Two individuals travelled further east into Pakistan reaching the Thar Desert or north-east Baluchistan. Three birds migrated in a south-westerly direction all the way and wintered in South-Iran. On average, the distance covered during migration was 2043±49 km (n = 21).

Houbara caught in China in the Eastern Jungar Basin and in the Gobi desert further east started their migration in a north-westerly and westerly direction. They avoided the Tien Shan Mountains and flew south-west along the same flyway as Central and East-Kazakh birds, around the Pamir. Two individuals from the Gobi desert were tracked throughout the winter, which they spent in East Turkmenistan, north-west of the Hindu-Kush (Fig. 1E). This is the northernmost wintering area for tracked birds. These two birds flew an average of 3810±174 km from their breeding area. Two birds from the Jungar Basin group (out of nine) also wintered in a nearby area at the Turkmen-Afghan border. All others however flew further south to South Iran or south-east and around the mountain range to Pakistan and the Thar Desert on the Indian border (Fig. 1D). Birds caught in the Jungar Basin travelled 3742±248 km (n = 9) on average.

A model investigating the effect of population and sex on distance travelled showed that the interaction of both factors had a significant effect (F_3_ = 12.5, P<0.0001). Clearly, population origin was the most important effect (F_4_ = 72, P<0.0001), but, for the Eastern Jungar population only, the effect of sex was also significant (t = 5.3, P<0.0001, Fig. 1D). Age (adult vs. juvenile) had no effect on the distance travelled (F_1_ = 1.3, P = 0.27) controlling for the effect of population origin.

Birds caught on the eastern side of the Arabian Peninsula in Oman and Yemen did not show migratory movements but occasionally made non-seasonal small-scale movements (only exceptionally larger than 100 km).

### Wintering ranges

**Figure 2:**
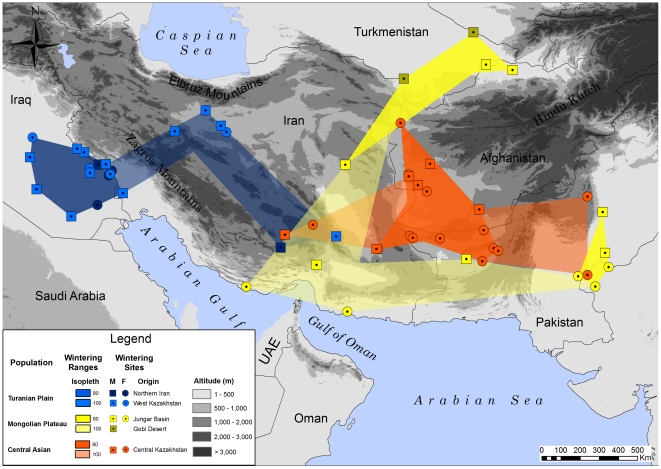
Wintering grounds of migrant Asian houbara bustards coming from breeding areas distributed across the greater part of the range.

Based on 80% LOCOH isopleths, wintering grounds were concentrated in four separate areas, (Fig. 2). On the western side of the Zagros Mountains, eastern Iraq hosted exclusively wintering birds from Northeast Caspian and Northern Iran. On the western and southern edge of the Hindu-Kush, an area of *ca*. 400,000 km^2^ mainly in Southwest Afghanistan and Northern Pakistan hosted wintering houbara originating from Central Kazakhstan. Chinese birds were found in two main zones in winter, one on the eastern edge of the Karakum desert in Turkmenistan and one in the Cholistan in Sindh (western Thar Desert, Pakistan).

These four core areas were extended when considering 100% LOCOH isopleths. Some Northeast Caspian and Northern Iran breeders did not cross the Zagros Mountains and wintered east of them in Central Iran. Birds from China were also found in southern Iran, with the 100% isopleth depicting a wintering range circumventing that of Central Kazakhstan birds by the north, west, south and east. These extended wintering ranges overlapped in South-Central Iran.

### Juveniles

**Figure 3:**
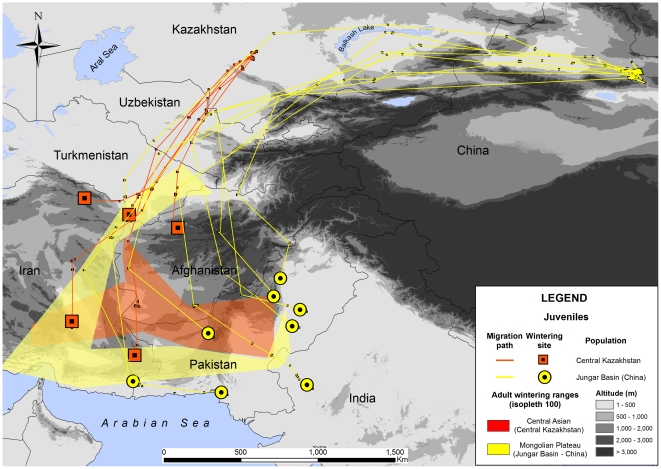
Migration paths and wintering ranges of juvenile Asian houbara bustards originating from Central Kazakhstan and Jungar Basin with reference to wintering ranges of adults.

Of 21 PTTs deployed on juveniles, 13 transmitted sufficient data to cover the autumn migration. This concerned eight birds from the Jungar Basin in China and five from Bedpak-Dala (Table 1). They migrated along the same migration paths as the adults in a given breeding area (Fig.3). Interestingly however, juvenile wintering ranges slightly differed from those of adults (Fig. 3). Three of the Central Kazakh juveniles wintered in Northeast Iran and Northwest Afghanistan, to the north of the observed adult wintering range. The two others wintered further south in Seistan and Kerman provinces (Iran), in what was defined above as a wintering range for Chinese birds. Similarly, six of eight from the Jungar Basin wintered slightly outside the adult wintering range. Of these 13 juveniles, only two completed one or more annual cycles, both returning to their initially selected wintering site.

## Discussion

The degree of migratory connectivity ascribed to populations is largely dependant on the scale that is chosen to define such populations and the areas they inhabit. We find that only very minor overlap is apparent between the wintering ranges of Western birds (West-Kazakhstan and Northern Iran) on one side and those of Central Kazakhstan and further east on the other. These pathways could be described as parallel migration [20], [29], [30], with both groups flying in a south-westerly direction, western birds avoiding the Caspian sea, and Eastern birds the mountain ranges of the Pamir and Hindu-Kush (Fig. 1). A migratory divide, possibly along the Aral Sea and mountain range in North-eastern Iran, could separate them. But birds need to be tracked from Turkmenistan and Northern Uzbekistan to explore this further. If our definition of populations or areas is limited to these two groups, then migratory connectivity is very strong indeed. However, birds caught in relatively small study areas (less than 100 km wide) were sometimes found wintering in areas distant by more than 1500 km (Fig. 1C & 1D). For example, Chinese birds caught in Mori (Fig. 1D) wintered north of the Hindu-Kush, in South Iran and on the Indian border. Viewed from this angle, connectivity seems weak and cannot be compared to the high levels of connectivity between breeding and wintering grounds described in some species [31].

Houbara tended to avoid major physical obstacles such as large water bodies or high mountains, preferring long detours inland and at low altitudes (maximum recorded *ca.* 2050 m a.s.l.). This strategy is common to many bird species that exclusively use flapping flight during migration [32], [33]. Moreover, houbara probably still make use of their historical immigration routes, as observed in other bird species [14], [34]. Of African origin, they are thought to have colonized central Asian habitats at the end of the last ice age [13] and have probably partly inherited their migration routes from the colonization process that progressed north from Arabia and Iran, bounded by the Caspian Sea to the West and the Hindu Kush and Pamir to the East.

It is interesting to note the small amount of overlap between the wintering ranges of far-eastern birds and those of the East and Central-Kazakh ones, the latter mainly spending the winter in an area corresponding to Northern Baluchistan and the border between Iran and Afghanistan. Although only two East-Kazakh birds were tracked throughout the winter, we obtained enough data from four others to know that they migrated to Afghanistan and eastern Iran and would have likely spent the winter there, or further south [35]. Segregation in wintering ranges is thus apparent between these two groups of birds (Fig. 2) but we recognize that a larger sample of birds could possibly affect the extent of this segregation. Indeed there is no migratory divide, both groups sharing similar flyways, travelling southwest through North Central Asia in order to avoid the mountain range.

South-central Iran, in particular Fārz, Kermān, Hormozgān and Seistan-Baluchistan provinces, appears to host a small number of individuals originating from western, central and eastern breeding populations (Fig. 2). Thus there is no single Iranian population as previously hypothesized [36]. Rather, birds in Iran are composed of breeding migrants in the north which predominantly spend the winter in Iraq, a winter population mainly in the south coming from many parts of the range, and a resident breeding population, little studied, but which is thought to belong to a wider group of residents also present in Baluchistan [37].

The natural prolongation to the south of this wintering range is the Arabian Peninsula and one would expect to find, in the eastern part of Arabia, a mixture of populations similar to that observed in South-Central Iran. A large number of Houbara used to winter on the Arabian Peninsula [38], [39], [40], [41] and a country like the U.A.E. does still host winterers, albeit in small numbers, originating from the greater part of the species' range in Asia, from the Northern Aral steppes to the Gobi desert [35], [41]. It is indeed striking that out of all the birds we tracked from Central Asia, not one wintered on the Arabian Peninsula. Recent and highly selective forces, in particular unregulated hunting associated with severe habitat degradation in Arabia are most likely the cause of this pattern [42], [43], [44], [45].

Juveniles followed very similar flyways to those of adults from the same region. Surprisingly however, they wintered mostly outside of adult wintering ranges. This would not seem to support the idea that they followed experienced adults all the way from the breeding to the wintering grounds on their first migration, as suggested for some diurnal migrants [46]. It is evident from our sample that birds from Kazakhstan flew shorter distances than those from China; thus migration in houbara may have evolved a genetic component determining migration direction and duration [47], but not an accurate map. Regarding natal dispersal, it is interesting that from only two chicks that survived more than one annual cycle (Chinese birds) both eventually returned to their natal area in spring, but only in their third spring, having spent their second spring and summer elsewhere (one of the chicks stayed in Uzbekistan). They also returned to their initially chosen wintering ranges in subsequent years. Information is generally lacking on the causes of the birds' death but it is known that many birds are caught on the wintering grounds; indeed trappers in Pakistan claimed the capture of two of the 13 juveniles that completed their outward migration.

Whether it is from habitat degradation or unregulated and excessive off-take, present declines or extinctions in certain parts of the range are a fact and future declines in some of the less affected populations are likely [11], [12], [48]. At a time when conservation measures are being implemented [49], precise knowledge of migratory movements and juvenile dispersal behaviour are critical. In this regard, this paper provides sound information that will assist in the identification of management units. But further work is required, notably on juvenile dispersal as well as on the basis of migratory behaviour — the extent to which it is genetically or socially determined. Displacement experiments for instance could be extremely useful [46], [50].
